# A retrospective analysis of taxane-based chemotherapy in small bowel adenocarcinoma

**DOI:** 10.1093/oncolo/oyag104

**Published:** 2026-03-23

**Authors:** Mir Lim, Nikhil Grandhi, Preksha Shah, Kanwal Raghav, Victoria Serpas, Maria Pia Morelli, Katrina Pedersen, Aparna Kalyan, Mohamed Salem, Ryan Huey, John Paul Shen, Huamin Wang, Hua Wang, Anjali Vinocha, Jane Thomas, Robert Wolff, Michael J Overman

**Affiliations:** Department of Gastrointestinal Medical Oncology, Division of Cancer Medicine, The University of Texas MD Anderson Cancer Center, Houston, TX 77030, United States; Department of Gastrointestinal Medical Oncology, Division of Cancer Medicine, The University of Texas MD Anderson Cancer Center, Houston, TX 77030, United States; Department of Gastrointestinal Medical Oncology, Division of Cancer Medicine, The University of Texas MD Anderson Cancer Center, Houston, TX 77030, United States; Department of Gastrointestinal Medical Oncology, Division of Cancer Medicine, The University of Texas MD Anderson Cancer Center, Houston, TX 77030, United States; Department of Gastrointestinal Medical Oncology, Division of Cancer Medicine, The University of Texas MD Anderson Cancer Center, Houston, TX 77030, United States; Department of Gastrointestinal Medical Oncology, Division of Cancer Medicine, The University of Texas MD Anderson Cancer Center, Houston, TX 77030, United States; Department of Medical Oncology, Mayo Comprehensive Cancer Center, Rochester, NY 55905, United States; Division of Hematology Oncology, Robert H. Lurie Comprehensive Cancer Center of Northwestern University, Chicago, IL 60611, United States; Department of Medical Oncology, Levine Cancer Institute, Atrium Health, Charlotte, NC 28204, United States; Department of Gastrointestinal Medical Oncology, Division of Cancer Medicine, The University of Texas MD Anderson Cancer Center, Houston, TX 77030, United States; Department of Gastrointestinal Medical Oncology, Division of Cancer Medicine, The University of Texas MD Anderson Cancer Center, Houston, TX 77030, United States; Department of Pathology, The University of Texas MD Anderson Cancer Center, Charlotte, NC 77030, United States; Department of Gastrointestinal Medical Oncology, Division of Cancer Medicine, The University of Texas MD Anderson Cancer Center, Houston, TX 77030, United States; Department of Gastrointestinal Medical Oncology, Division of Cancer Medicine, The University of Texas MD Anderson Cancer Center, Houston, TX 77030, United States; Department of Gastrointestinal Medical Oncology, Division of Cancer Medicine, The University of Texas MD Anderson Cancer Center, Houston, TX 77030, United States; Department of Gastrointestinal Medical Oncology, Division of Cancer Medicine, The University of Texas MD Anderson Cancer Center, Houston, TX 77030, United States; Department of Gastrointestinal Medical Oncology, Division of Cancer Medicine, The University of Texas MD Anderson Cancer Center, Houston, TX 77030, United States

**Keywords:** taxane, SBA, TP53, chemotherapy

## Abstract

**Background:**

Small bowel adenocarcinoma (SBA) is molecularly distinct from colorectal and gastric cancers, yet treatment typically parallels colorectal cancer. We evaluated the activity of taxane-based therapy in the largest SBA cohort to date.

**Methods:**

We retrospectively reviewed SBA patients treated with taxane-based chemotherapy at MD Anderson Cancer Center from 1994 to 2024. Eligible patients had pathologic confirmation, received >1 treatment cycle, and had tumor response evaluation. Survival analyses were analyzed using Kaplan–Meier and Cox proportional hazard models.

**Results:**

Seventy patients were identified. Median age was 57, and 59% were male. Primary sites were duodenum (44%), jejunum (34%), and ileum (16%). Metastatic sites included peritoneum (39%) and liver (31%). Common mutations were *TP53* (63%), *KRAS* (47%), *SMAD4* (24%), and *APC* (15%). Taxanes were administered as single agents (29%) or in combination (71%), most often in second- (40%) or third-line (33%) settings. Overall response rate was 24%. Median time to progression (mTTP) was 3.1 months (95% CI: 2.0-4.2) and median overall survival (mOS) was 8.7 months (95% CI: 7.4-10.1). Efficacy did not differ by treatment line, regimen type, or tumor site, but was significantly associated with *TP53* status; response rate was 20% in *TP53*-mutated vs 45% in wild-type (*P *= .009), with mTTP 2.5 vs 4.9 months (*P *= .009) and mOS 7.3 vs 10.6 months (*P *= .002). On multivariable analysis, *TP53* mutation predicted worse outcomes.

**Conclusion:**

Taxane-based therapy demonstrated activity in metastatic SBA, with 24% response and 3.1-month mTTP. *TP53* mutation may be a negative predictive marker for taxane efficacy. These findings support prospective investigation in metastatic SBA.

Implications for Practice This is the largest cohort of patients with metastatic SBA treated with taxane-based chemotherapy. Taxane-based chemotherapy demonstrates a meaningful clinical benefit with a response rate of 24%, median TTP of 3.1 months, and median OS of 8.7 months. *TP53* mutations may represent a negative predictive marker for taxane-based chemotherapy.

## Introduction

Small bowel adenocarcinoma (SBA) is a rare cancer accounting for approximately 40% of small bowel malignancies.^1^ According to the Surveillance, Epidemiology, and End Results (SEER) program, around 12 440 new cases of small intestine cancer are diagnosed in 2024 and incidence of new cases has increased from 1.5/100 000 people in 2000 to 2.6/100 000 people in 2021.^2^ Around 27% of patients present with metastatic disease and have a 5-year overall survival (OS) of 3% to 19%.^1^^,^^3–5^

Given the rarity of the disease, most of the evidence for treatment comes from either small, single-arm clinical trials or retrospective analyses. Due to the dearth of evidence, clinical management of SBA mirrors treatment paradigms of colorectal cancer (CRC). Much like CRC, current first-line therapy for metastatic SBA includes fluoropyrimidine plus platinum-based chemotherapy based on 2 phase II trials.^6^^,^^7^ Treatment options in the second-line setting and beyond are also extrapolated from data for CRC. However, a recent comprehensive molecular analysis of SBA showed that SBA is a distinct genomic entity; specifically, SBA exhibits significantly different rates of *APC, CDKN2A,* and *BRAF*-V600E mutations compared to CRC; higher rates of KRAS mutations compared to gastric carcinomas; and enrichment of *HER2* mutations, microsatellite instability (MSI), and high tumor mutational burden.^8^ This demonstrates the need for an individualized approach to SBA treatment, including therapeutic classes not commonly utilized in CRC.

Taxane-based chemotherapy plays a limited role in CRC treatment but has been shown to have activity in SBA. A phase II study of 13 SBA patients showed that nab-paclitaxel had higher response rates and longer progression-free survival (PFS) in SBA compared to CpG island methylator phenotype-high CRC.^9^ In our prior retrospective analysis of 20 patients treated with taxane-based therapy, 30% of patients with SBA were found to have tumor response and 35% of patients had stable disease. Median time to progression (TTP) on taxane-based therapy was 3.8 months and OS was 10.7 months.^10^

Currently, taxane-based chemotherapy is listed as a category 2A recommendation in the NCCN guidelines for second-line treatment option in the unresectable/metastatic disease setting.^11^ While molecular testing has transformed the treatment of other cancers in the advent of targeted therapy, its role in SBA remains limited, with little evidence to guide its use beyond assessing MSI. To better characterize the activity of taxane-based therapy and the unique molecular profile of SBA, we conducted an updated retrospective analysis of 70 patients with SBA and describe the role of *TP53* mutation status as a potential predictive biomarker to guide therapy.

## Methods

### Design and patient population

We conducted a retrospective study of patients with locally advanced or metastatic SBA treated with taxane-based chemotherapy at the University of Texas MD Anderson Cancer Center (MDACC) between 1994 and 2024. This retrospective analysis was approved by the Institutional Review Board of MDACC. Patients with SBA were identified from the MDACC tumor registry and a total of 424 locally advanced or metastatic SBA patients were identified. Eligible patients had pathologic confirmation of SBA, received at least one cycle of a taxane-based regimen, and had radiographic tumor response evaluation. Patients enrolled in a clinical trial or those with primary ampullary adenocarcinoma were excluded.

Baseline characteristics of the study population, including age, gender, ethnicity, site of primary lesion, predisposing conditions, histologic grade, genomic alterations, and sites of metastases, were collected from the medical record. MSI was determined by standard of care immunohistochemical or PCR-based testing, or next-generation sequencing.

For each episode of taxane-based therapy use, we performed a detailed review of the treatment history, including performance status at time of treatment initiation, prior lines of therapy, specific taxane-based regimens used, and best response to taxane-based therapy per medical record review. Prior lines of therapy were defined by the number of chemotherapy and/or targeted agents that were administered for advanced or metastatic disease. Systemic therapy for metastatic disease started within 12 months of adjuvant therapy was considered as second-line treatment.

To evaluate the impact of *TP53* mutation status on the efficacy of oxaliplatin- or irinotecan-based chemotherapy, a separate cohort of metastatic SBA patients with known *TP53* status treated with either irinotecan-based (*N* = 94) or oxaliplatin-based chemotherapy (*N* = 109) in either first- or second-line setting was identified. TTP and overall survival (OS) were calculated for patients who received oxaliplatin-based therapy or irinotecan-based therapy from both cohorts.

We defined TTP as the time from treatment initiation to evidence of radiographic disease progression. Radiographic response was retrospectively assessed by the treating physician at first-available post-treatment imaging and categorized as response, stable disease, or progression. OS was defined as the time from treatment start until death or last follow-up. Patients who were alive at last follow-up were censored.

### Statistical analysis

Clinical and demographic characteristics of the study population were summarized using descriptive statistics. Median TTP and median OS were determined using Kaplan–Meier analysis. Log-rank of 2-sided *P* values < .05 were considered statistically significant. A multivariable Cox proportional hazards analysis was also done to evaluate the association between selected covariates and TTP/OS; covariates included *TP53* mutation status (yes vs no), *KRAS* mutation (yes vs no), location of primary tumor (duodenum vs other), taxane therapy (single-agent vs combination), type of taxane therapy (paclitaxel vs nab-paclitaxel vs docetaxel), Eastern Cooperative Oncology Group performance status (0 vs ≥1), and prior lines of therapy (0-1 vs ≥2). Statistical analyses were conducted using SPSS version 24.

## Results

### Patient characteristics

Seventy SBA patients treated with taxane-based therapy were identified. Demographic characteristics of patients are presented in Table 1. The median age at diagnosis was 57 years (range 33-80 years), with the majority of patients being male (59%). Primary site was 44% duodenum, 34% jejunum, and 16% ileum. The most common presenting metastatic site was the peritoneum (39%) followed by the liver (31%). Most patients (86%) had a proficient mismatch repair tumor. Mutation status for *TP53*, *KRAS*, *SMAD4*, *BRAF*, and *APC* were available in 55, 70, 55, 58, and 40 patients, respectively, with mutation frequencies of 63%, 47%, 24%, 10%, and 15%, respectively.

**Table 1:** Patients’ baseline characteristics.

Characteristic	*N* (%)
**Median age at diagnosis (range), year**	57 (33-80)
**Gender**	
** Male**	41(58.6%)
** Female**	29 (41.4%)
**Race**	
** White**	55 (78.6%)
** Black**	10 (14.3%)
** Asian**	1 (1.4%)
** Other**	4 (5.7%)
**Primary lesion site**	
** Duodenum**	31 (44.3%)
** Jejunum**	24 (34.3%)
** Ileum**	11 (15.7%)
** Site unknown**	4 (5.7%)
**Primary resected**	
** Yes**	53 (75.7%)
** No**	17 (24.3%)
**Predisposing conditions**	
** Inflammatory bowel disease**	8 (11.4%)
** Celiac disease**	1 (1.4%)
** Lynch syndrome**	2 (2.9%)
** Familial adenomatous polyposis**	1 (1.4%)
**Histologic grade**	
** Well**	1 (1.4%)
** Moderate**	37 (52.9%)
** Poor**	30 (42.9%)
** Unknown**	2 (2.9%)
**Presenting metastatic sites**	
** Liver**	22 (31.4%)
** Peritoneum**	27 (38.6%)
** Liver + peritoneum**	7 (10.0%)
** Multiple and other Mets**	11 (15.7%)
** Locally advanced**	3 (4.3%)
**MMR status**	
** pMMR**	60 (85.7%)
** dMMR**	3 (4.3%)
** Unknown**	7 (10.0%)
** Molecular**	Mutated
** *KRAS* (*n* = 70)** ^a^	33 (47.1%)
** *TP53* (*n* = 55)**	35 (63.6%)
** *BRAF* (*n* = 58)** ^b^	6 (10.3%) (Non-*V600E*)
** *SMAD4* (*n* = 55)**	13 (23.6%)
** *APC* (*n* = 40)**	6 (15%)
** *NGS panel***	
** *Single gene panel***	2 (2.86%)
** *46-70 gene panel***	25 (35.71%)
** *>300 gene panel***	35 (50%)
** *Not done***	8 (11.42%)

a KRAS mutations—G12D = 11; G12V = 9; G12A= 3; other mutations (G12R, G13A, G13D, G2A, Q16H) = 7, unknown = 3.

b BRAF mutations—D594G = 2; D594N = 1; G469A = 1; G569A = 1; K601E = 1.

### Patterns of taxane-based therapy administration

As detailed in Table 2, taxane-based therapy was administered as single agent nab-paclitaxel, paclitaxel, or docetaxel in 29% of patients. The combination of gemcitabine and taxane was given in 61% of patients. Other taxane-based combinations were given in 10% of patients. Treatment with taxane-based therapy was given in the metastatic setting as second-line, third-line, and fourth-line or greater in 40%, 33%, and 20% of patients, respectively.

**Table 2:** Characteristics of taxane-based regimens used.

Characteristic	*n* (%)
**Median age at start of taxane therapy (range), years**	60 (34-83)
**Single agent vs combination**	
**Single agent**	20 (28.6%)
**Gemcitabine-based combinations**	43 (61.4%)
**Other combinations^a^**	7 (10%)
**Taxane type**	
** Paclitaxel**	31 (44.3%)
** Nab-paclitaxel**	32 (45.7%)
** Docetaxel**	7 (10%)
**Baseline ECOG performance score**	
** 0**	23 (32.9%)
** 1**	40 (57.1%)
** 2**	2 (2.9%)
** 3**	1 (1.4%)
**Not available**	4 (5.7%)
**Prior line(s) of therapy**	
** 0**	5 (7.1%)
** 1**	28 (40.0%)
** 2**	23 (32.9%)
** 3-4**	14 (20%)
**Best response**	
** Response**	17 (24.3%)
** Stable**	18 (25.7%)
** Progression**	35 (50%)

a Other combinations—5 FU based chemo; platinum-based chemo; 5FU + platinum based chemo; bevacizumab, pembrolizumab, ramucirumab.

### Response to treatment and outcomes

Seventeen patients (24%) achieved response, 26% achieved stable disease, and half of the patients were found to have progression of disease as best response to taxane-based therapy. No significant correlations were found between response to taxane therapy and site of first metastasis, number of metastatic sites, single vs combination taxane therapy, tumor grade, resection of primary tumor, prior lines of therapy, and taxane group. Median OS was significantly longer for patients who had stable disease or partial response compared to those who had progression of disease (10.3 months vs 6.4 months) (*P *= .01).

**Figure 1.**
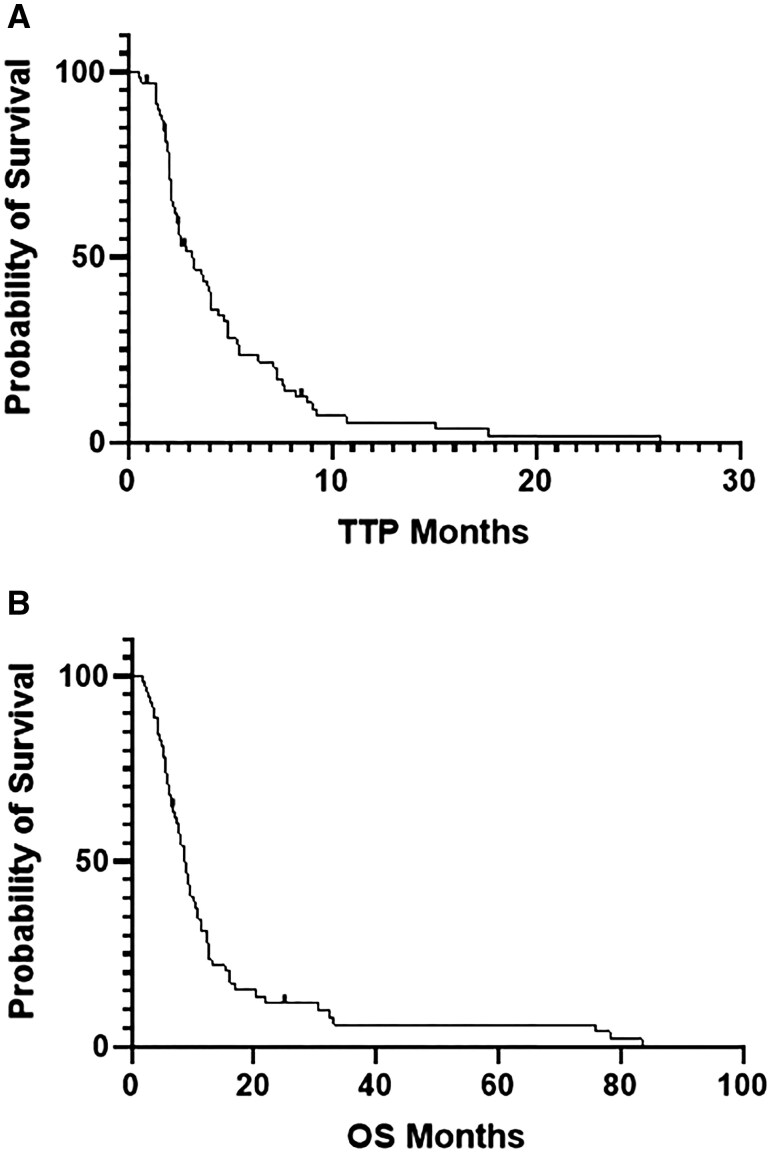
Kaplan–Meier survival curves for patients with SBA treated with taxane-based therapy. (A) Time to progression. (B) Overall survival.

**Figure 2.**
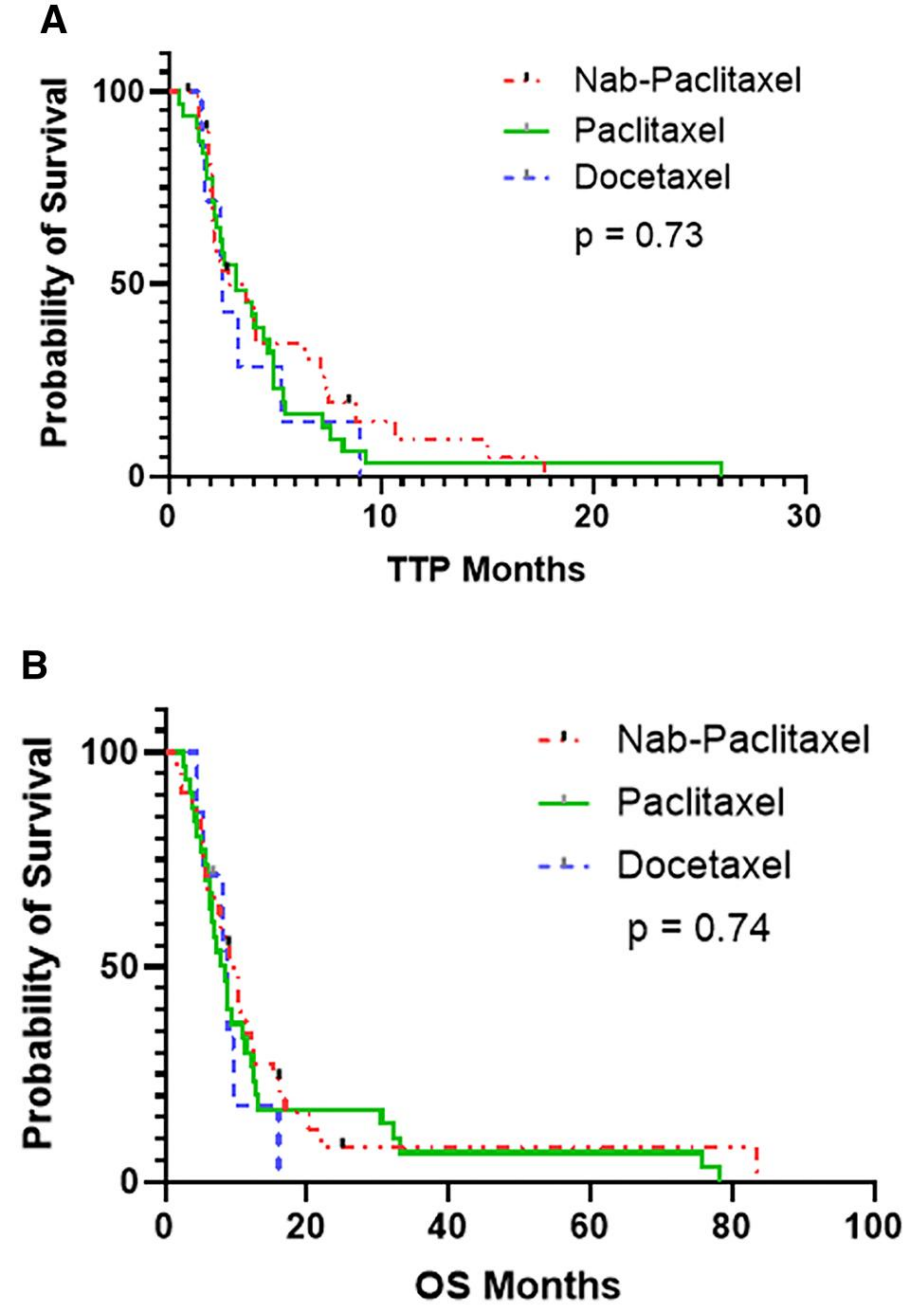
Kaplan–Meier survival curves for patients with SBA treated with taxane-based therapy stratified by taxane group. (A) Time to progression. (B) Overall survival.

**Figure 3.**
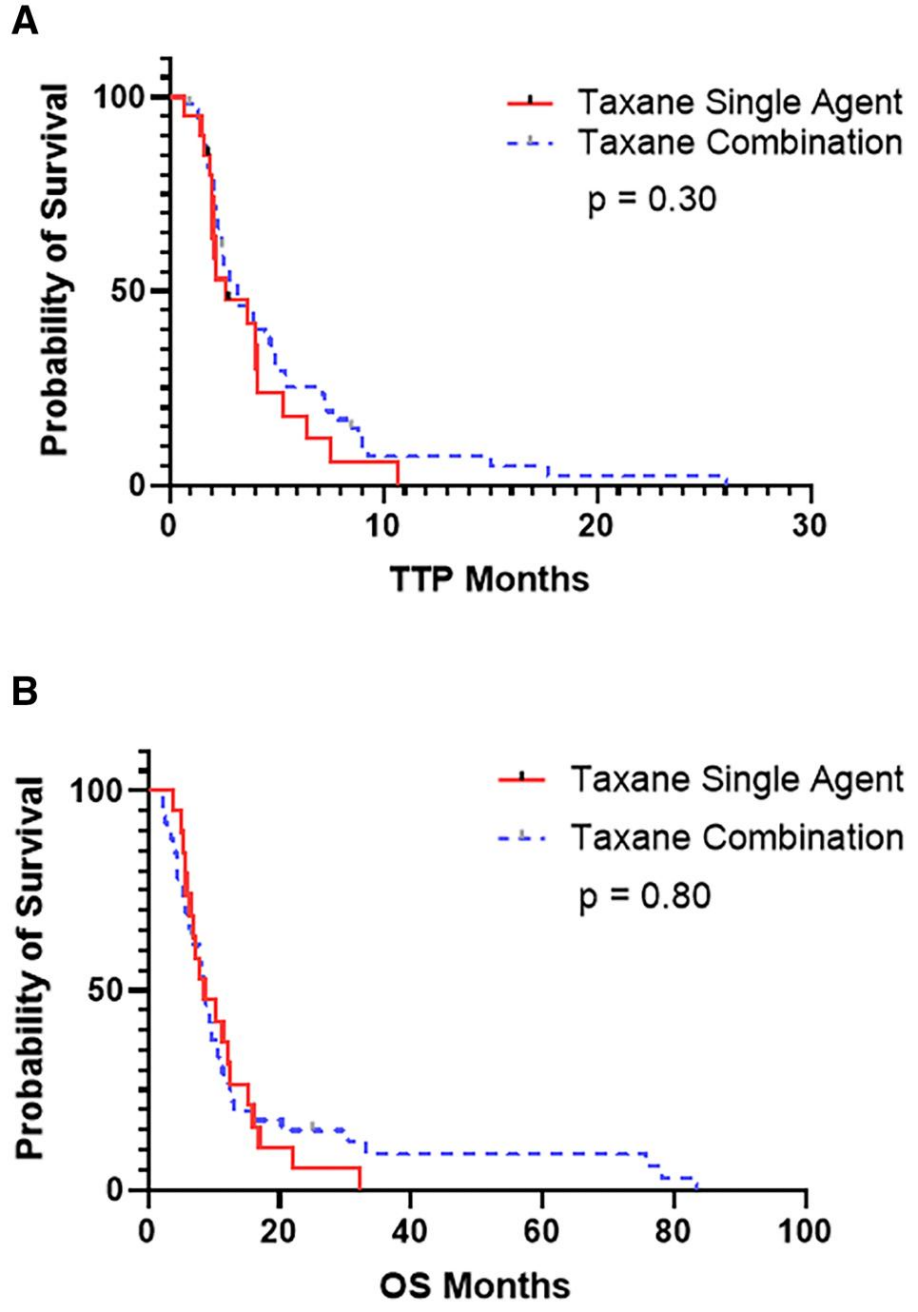
Kaplan–Meier survival curves for patients with SBA treated with taxane-based therapy stratified by single vs combination-based therapy. (A) Time to progression. (B) Overall survival.

Median TTP was 3.1 months (95% CI: 2.0-4.2 months), and median OS was 8.7 months (95% CI: 7.4-10.1 months, Figure 1). There was no significant difference in median TTP or OS regardless of type of taxane-based therapy administered (Figure 2), and no difference was seen whether treatment was given as single agent taxane-based therapy (2.6 months) vs a gemcitabine-based combination (2.5 months) (*P *= .75, Figure 3). Furthermore, TTP and OS did not differ by lines of therapy (0-1 lines of therapy [*n* = 33] vs ≥2 lines of therapy [*n* = 37]—median TTP of 2.82 vs 3.64 months, *P *= .642; median OS of 9.22 vs 8.76 months, *P *= .90; Figure S1, see online supplementary material for a color version of this figure), primary tumor site, or *KRAS, SMAD4*, or *APC* mutational status. In the multivariable analysis, *TP53* mutation status, compared to wild-type, was associated with a statistically significant reduction in TTP (HR 2.23, 95% CI 1.03-4.81, *P = *.042) and OS (HR 2.66, 95% CI 1.30-5.46, *P = *.001). No other significant associations were observed (Table S1).

### TP53-mutation status and efficacy

**Figure 4.**
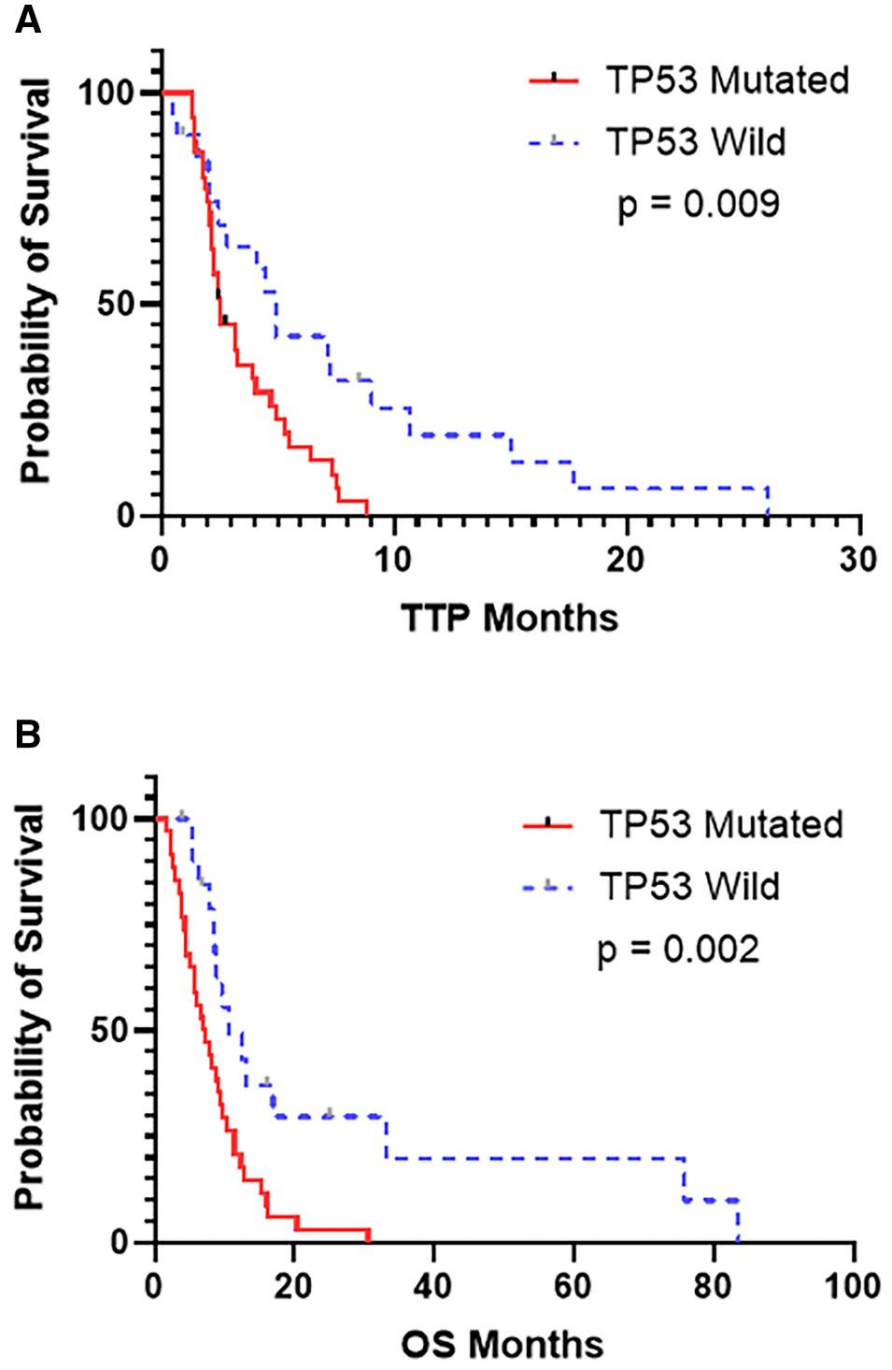
Kaplan–Meier survival curves for patients with SBA treated with taxane-based therapy stratified by TP53 mutation status. (A) Time to progression. (B) Overall survival.

Interestingly, efficacy differed by *TP53* mutation status with *TP53*-mutated cases showing a 20% response vs wildtype cases showing a 45% response (*P *= .009). Median TTP was significantly longer in tumors that did not have a *TP53* mutation, with *TP53*-mutated cases having a TTP of 2.5 months vs a TTP of 4.9 months in wildtype cases (*P *= .009, Figure 4). Furthermore, median OS significantly differed between patients with *TP53*-mutated vs *TP53*-wildtype SBA (7.3 months vs 10.6 months) (*P *= .002).

To further evaluate whether the predictive value of *TP53* mutation status was specific to taxane-based therapy, we analyzed 2 combination cohorts of metastatic SBA patients treated with or without taxane therapy and known *TP53* status. *TP53* mutation status was not found to have a statistically significant impact on TTP or OS in those who received oxaliplatin-based chemotherapy (*N* = 109; *P* = .23 and .11, respectively). For patients treated with irinotecan-based chemotherapy (*N* = 94), median TTP was not significantly impacted by TP53 mutation status (*P* = .08), but median OS was significantly worse for patients with TP53 mutations (*P* = .01; Figures S2 and S3, see online supplementary material for a color version of these figures).

## Discussion

This cohort of 70 SBA patients treated with taxane-based chemotherapy is the largest to date and provides support for the emerging evidence on the role of taxane-based chemotherapy in this disease. The response rate of 24%, median TTP of 3.1 months, and median OS of 8.7 months demonstrate a modest clinical benefit in refractory metastatic SBA patients. For a rare cancer with limited treatment options, our findings are directly relevant for guiding clinical care.

Our results show comparable clinical activity across lines of therapy, although 93% of the 70 patients in this study received taxane-based chemotherapy in the second line or higher setting. Interestingly, our data suggest that *TP53* mutation may represent a negative predictive biomarker for response to taxane-based therapy in SBA.

The findings reported here are consistent with our earlier retrospective study of 20 patients with SBA who were treated with taxanes.^10^ They also align with results from a small phase II prospective trial of 10 evaluable patients treated with nab-paclitaxel, which reported a median PFS of 3.2 months and OS of 10.9 months.^9^ Comparable outcomes have been observed with other second-line therapies, such as a retrospective multicenter study of 51 patients treated with FOLFIRI after progression on first-line platinum-based chemotherapy demonstrating a median PFS, OS, and ORR of 3.2 months, 10.5 months, and 20%, respectively.^12^

Given the limited evidence, even less is known about specific taxane regimens and their associated responses. Our study found that patients treated with single-agent taxanes (nab-paclitaxel, paclitaxel, or docetaxel) demonstrated similar TTP compared to patients treated with gemcitabine-based combination therapy. This finding suggests that the addition of gemcitabine may offer minimal benefit. However, given the limited sample size, our analysis may be underpowered to detect a benefit, and our results should not be interpreted as definitive evidence against the potential value of combination-based therapies. Prospective studies are needed to confirm whether it can be omitted or combined with alternative agents to improve safety without compromising efficacy. We await the results of an ongoing clinical trial (NCT04205968) comparing response to ramucirumab and paclitaxel vs FOLFIRI in the second-line setting.

With the advent of molecular testing, it is essential to incorporate patient-specific genomic findings to optimize therapy. However, the biomarkers/targets are very limited in patients with SBA. Mismatch repair status has been used to guide the use of immunotherapy—a prospective trial showed an ORR of 42.1% and a median TTP of 9.2 months in 19 SBA patients treated with pembrolizumab.^13^ A retrospective study from our institution highlighted the lack of activity of anti-EGFR therapy in SBA, even in RAS-wild type patients.^14^ Approximately 40%-48% of patients with SBA harbor a *TP53* mutation and its presence has been previously shown to be indicative of poor overall survival, making it an important marker for therapeutic implications.^15–17^ In our study, *TP53* status was known in 53 patients, of which 34 were mutated; we observed that patients with *TP53* mutations had a statistically significant inferior response to treatment with taxanes compared to patients without TP53 mutations.

*TP53* mutation status and sensitivity to taxane therapy have been studied in other cancers with varying conclusions. In a phase 3 study of patients with locally advanced, large operable, or inflammatory breast cancers randomly assigned to an anthracycline or taxane-based regimen, *TP53* status was found to be prognostic for overall survival but not predictive of response to taxanes.^18^ Similarly, the presence of a *TP53* mutation was associated with poorer prognosis but showed no predictive value for sensitivity to docetaxel in a randomized phase III trial for node-positive breast cancer treated with or without adjuvant docetaxel in combination with doxorubicin-based chemotherapy.^19^ In ovarian cancer, mutant *TP53* tumors showed significantly higher response rates to paclitaxel-based chemotherapy compared to wild-type p53 tumors.^20^ However, a preclinical study suggests that *TP53* inactivating mutational signatures are associated with resistance to taxanes in ovarian cancer cells.^21^ In metastatic non-small-cell lung cancer, *TP53* mutations did not affect response to single agent paclitaxel.^22^ The variable effects of TP53 mutations on taxane sensitivity could be due to differences in microtubule regulation, mutation-specific TP53 function, and tissue-specific signaling pathways, though conflicting studies highlight the incomplete understanding of the underlying pathophysiology.^23–25^ Specifically in SBA, little is known about the role of *TP53* mutation status as a biomarker of response to chemotherapy. Our results suggest chemoresistance to taxane-based therapy in TP53-mutated SBA and supports further investigation in other datasets.

Lastly, given that *APC* mutations are uniquely enriched in SBA, we explored whether it might serve as a predictive marker for response to taxane therapy, especially given prior in vitro data suggesting an association of *APC* mutation status and taxane resistance in CRC.^9^ In our analysis, *APC* mutation was not associated with a statistically significant difference in either TTP (5.3 months vs 2.7 months, *P *= .346) or OS (16.5 months vs 8.1 months, *P *= .127) among patients treated with taxane-based therapy.

We acknowledge that our study has several limitations. The retrospective, single-institution nature of the study limits the overall generalizability of our findings. Moreover, it is difficult to standardize response assessment and timing of response as this was assessed by the treating physician. The treatment cohort also spans a 30-year treatment period, during which non-treatment related factors—such as advances in supportive care that improve chemotherapy tolerability, enhanced imaging techniques that enable more precise response assessment, and increasingly personalized approaches to care—may have introduced confounding variables.

## Summary

In summary, this is the largest study to report on efficacy of taxane therapy in metastatic SBA. Our study suggests that taxane-based regimens may have a role in treatment of patients with metastatic SBA. In addition, our study demonstrates the potential value of *TP53* mutation in predicting tumor response to taxane-based therapy. Future prospective studies are needed to confirm our findings in patients with metastatic SBA.

## Supplementary Material

oyag104_Supplementary_Data

## Data Availability

Deidentified patient data from the clinical cohort will be provided to interested parties upon request.
