# Supplementary material for: A retrospective analysis of taxane-based chemotherapy in small bowel adenocarcinoma
Source: Oncologist. 2026 Mar 23;31(5):oyag104. doi: 10.1093/oncolo/oyag104 (PMC13076934; doi:10.1093/oncolo/oyag104)
Supplement: oyag104_Supplementary_Data [file oyag104_supplementary_data.docx]

|  | TTP | | | | OS | | | |
| --- | --- | --- | --- | --- | --- | --- | --- | --- |
| Variable | HR | 95% CI HR | | P-value | HR | 95% CI HR | | P-value |
| *TP53* (mutated vs. wild-type) | 2.246 | 1.016 | 4.963 | 0.046 | 2.738 | 1.288 | 5.818 | 0.009 |
| *KRAS* (mutated vs. wild-type) | 1.138 | 0.566 | 2.284 | 0.717 | 0.817 | 0.424 | 1.575 | 0.547 |
| Primary tumor site (other vs. duodenum) | 0.655 | 0.327 | 1.313 | 0.233 | 0.755 | 0.390 | 1.461 | 0.405 |
| Taxane therapy (combination vs. single-agent) | 0.680 | 0.316 | 1.463 | 0.324 | 0.716 | 0.320 | 1.603 | 0.417 |
| Chemotherapy regimen |  |  |  |  |  |  |  |  |
| Paclitaxel vs. Nab-paclitaxel | 1.517 | 0.714 | 3.225 | 0.279 | 1.384 | 0.626 | 3.059 | 0.422 |
| Docetaxel vs. nab-paclitaxel | 1.134 | 0.388 | 3.316 | 0.819 | 1.010 | 0.283 | 3.601 | 0.988 |
| ECOG (≥1 vs. 0) | 1.058 | 0.534 | 2.098 | 0.872 | 0.935 | 0.448 | 1.952 | 0.857 |
| Prior lines of therapy (≥2 vs. 0-1) | 0.918 | 0.467 | 1.806 | 0.805 | 1.023 | 0.519 | 2.017 | 0.947 |
| Era of treatment (Post 9/2/2019 vs. Pre 9/2/2019) | 1.684 | 0.866 | 3.274 | 0.125 | 1.835 | 0.911 | 3.699 | 0.089 |

**Supplementary Table 1: Multivariable Cox proportional hazards analysis of median TTP and OS among patients with SBA treated with taxane-based therapy**

A.


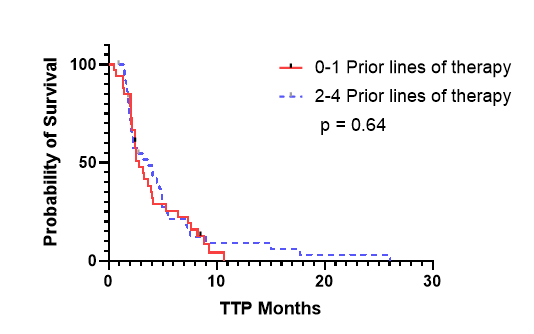

B.


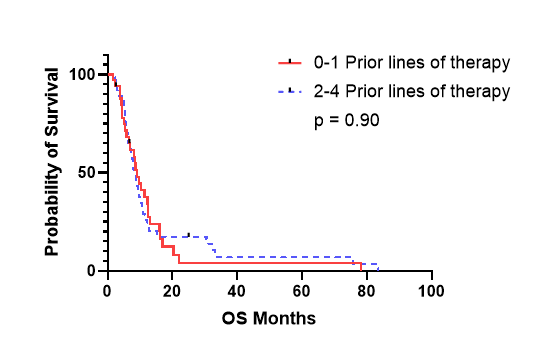

**Supplementary Figure 1: Kaplan-Meier survival curves for patients with SBA treated with taxane-based therapy stratified by number of prior lines of therapy. A: Time to progression for 0-1 (N=33) vs. 2-4 (N=37) prior lines of therapy (2.82 vs. 3.64 months, p=0.642). B: Overall survival for 0-1 vs. 2-4 prior lines of therapy (9.22 vs. 8.76 months, p=0.90).**

A.


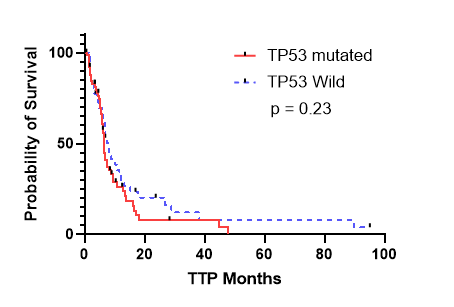


B.


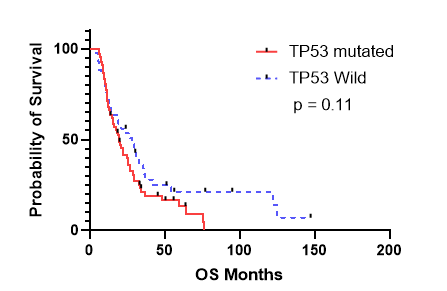


**Supplementary Figure 2: Kaplan-Meier survival curves for patients with SBA treated with oxaliplatin-based therapy (combined cohort of patients treated with and without taxanes, N=109) with known TP53 mutation status. A: Time to progression for TP53-mutated vs. TP53-wild-type (6.46 vs. 7.87 months, p=0.23). B: Overall survival for TP53-mutated vs. TP53-wild-type (19.82 vs. 28.22 months, p=0.11).**

A.


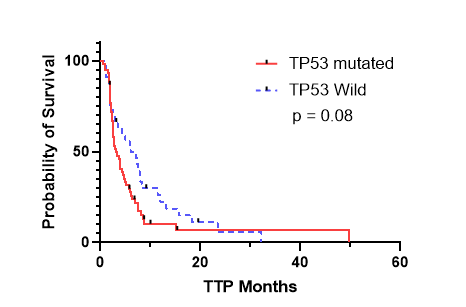


B.


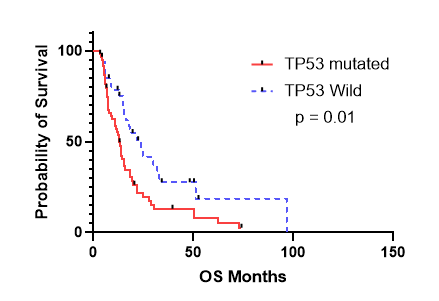


**Supplementary Figure 3: Kaplan-Meier survival curves for patients with SBA treated with irinotecan-based therapy (combined cohort of patients treated with and without taxanes, N=94) with known TP53 mutation status. A: Time to progression for TP53-mutated vs. TP53-wild-type (3.11 vs. 6.10 months, p=0.08). B: Overall survival for TP53-mutated vs. TP53-wild-type (12.99 vs. 23.89 months, p=0.01).**
